# 

**Published:** 1898-03

**Authors:** 


### The American Medical Association.

*Section on Materia Medica and Therapeutics.*—The following papers and discussions have been promised for the meeting at Denver, Col., June 7-10, 1898:

"Yellow Fever: Its Etiology and Treatment." Discussion by Surgeon-General George M. Sternberg, M. D., of Washington, D. C.; Prof. John Guiteras, M. D., of Philadelphia; Sollace Mitchell, M. D., of Jacksonville, Fla.; T. S. Scales, M. D., of Mobile, Ala.; C. B. Thornton, M. D., of Memphis, Tenn.; H. M. Bracken, M. D., of Minneapolis, Minn.; P. E. Archinard, M. D., of New Orleans, La.

"Aims of Modern Treatment of Tuberculosis." By Prof.

Edwin Klebs, M. D., of Chicago. Discussion by Charles Denison, M. D., of Denver, Col.; C. H. Whitman, M. D., of Los Angeles, Cal.

"Serum Therapy of Tuberculosis." By Prof. S. O. L. Potter, M. D., of San Francisco, Cal. Discussion by Prof. James M. Anders, M. D., of Philadelphia.

"The Therapeutics of Pulmonary Phthisis." By Paul Paquin, M. D., of St. Louis, Mo.

"Tuberculin as a Diagnostic and Curative Agent, with Report of 250 Tubercular Cases Treated." By C. H. Whitman, M. D., of Los Angeles, Cal.

"The Practical Value of Artificial Serum in Medical Cases." By P. C. Remondino, M. D., of San Diego, Cal.

"The Use of Remedies in Diseases of the Heart and Blood-vessels." By T. Lauder Brunton, M. D., D. Sc., F. R. S., London, England.

"The Mescal Button." By Prof. D. W. Prentiss, M. D., of Washington, D. C.

"The Modern Intestinal Antiseptics and Astringents." By William Frankhauser, M. D., of New York.

"To What Extent is Typhoid Fever Favorably Modified in its Course, Duration, Termination or Sequelæ by the Administration of Drugs." By Frank Woodbury, M. D., of Philadelphia, Pa.

"Strychnine." By J. N. Upshur, M. D., of Richmond, Va.

"Methods of Teaching Materia Medica and Tharapeutics." By Prof. G. H. Rohe, M. D., of Baltimore.

"The Study of Materia Medica and Therapeutics." By H. M. Bracken, M. D., of Minneapolis, Minn.

"The Great Therapeutic Importance of Rational Adaptation of Cathartic Remedies to the Physiological Functions of the Gastro-intestinal System." By E. D. McDaniels, M. D., LL.D., of Mobile, Ala.

"Why the Pharmacopœial Preparations Should be Prescribed and Used by the Profession." By Leon L. Solomon, M. D., of Louisville, Ky.

"The Use of Electricity by the General Practitioner." By Caleb Brown, M. D., of Sac City, Ia.

The following have also promised papers, subjects to be announced very soon, together with the day assigned for each discussion and paper:

Dr. I. E. Atkinson, of Baltimore, Md.; Dr. Henry Beates, of Philadelphia, Pa.; Dr. T. M. Balliet, of Philadelphia, Pa.; Dr. George F. Butler, of Chicago, Ill.; Dr. Dudley W. Buxton, of London, Eng.; Dr. J. Solis-Cohen, of Philadelphia, Pa.; Dr. N. S. Davis, Jr., of Chicago, Ill., Dr. P. J. Farnsworth, of Clinton, Ia.; Dr. J. E. Moses, of Kansas City, Mo.; Prof. Joseph Remington, of Philadelphia, Pa.; Dr. L. E. Sayre, of Lawrence, Kan.; Dr. H. V. Sweringen, of Fort Wayne, Ind.; Dr. E. L. Stephens, of Fort Worth, Texas.

The chairman will be pleased to receive and place upon the program subjects for discussion and papers.

John V. Shoemaker, M. D., Chairman,
1519 Walnut St., Philadelphia, Pa.

---

### Texas State Medical Association—Announcement.

Thirtieth Annual Meeting of the Texas State Medical Association, April 26th, 27th, 28th, 29th, 1898, Houston, Texas.

Dear Doctor:—As Chairman of the Committee of Arrangements, I desire to call the attention of the medical profession throughout the State, as well as the members of the Texas State Medical Association, to the coming meeting of the latter in Houston, 26th, 27th, 28th and 29th of April, 1898.

While the outlook for a large attendance is excellent, yet each member of the Association and profession should make this meeting a personal matter; come and attend; and use his influence to interest his doctor-neighbors in the meeting also.

The committee have made ample and reasonable arrangements with hotels and private boarding houses for the accommodation of those in attendance.

An unusually interesting programme has been arranged by your committee. The chairmen of the different sections are working assiduously for a large number of papers.

"Questions of the most vital import, not only to every physician, but to every citizen of the State as well, will come before this meeting. Houston is accessible to all, a delightful place to meet."

The committee may be able to secure a rate of one fare for round trip, if not the rate of one and a third fare, on the certificate plan, will prevail. Fraternally yours,

Joseph A. Mullen, M. D.,
March 1st, 1898. Chirman Com. of Arrangements.

HALLETTSVILLE, TEXAS, Feb. 26, 1898.

*Editor Texas Medical Journal:*

DEAR DOCTOR:—Please make notice in your valuable journal of the following:

Thursday, February 24th, there assembled at the office of Dr. Brewer, at Hallettsville, all the practicing physicians of Hallettsville, for the purpose of organizing Lavaca County Medical Society. Those present were: Drs. A. F. Newbury, J. E. Lay, C. H. Brewer, A. A. Ledbetter, T. R. Knox and Louis Hirschfeld. Dr. Lay was elected president pro tem., and Dr. Brewer secretary pro tem. It was decided to call a meeting of all practicing physicians of Lavaca County on Tuesday, March 8th, at 1 o'clock p. m., at the opera house building, for the purpose of forming a permanent organization. The society has issued a call to all the physicians of the county.

Respectfully,

C. H. BREWER, M. D., Sec'y pro tem.

